# Supplementary material for: Superior cardiovascular protection with GLP-1 RAs over SGLT2 inhibitors in DM and HFpEF: A propensity score matching study
Source: PLoS One. 2025 Jun 26;20(6):e0326534. doi: 10.1371/journal.pone.0326534 (PMC12200838; doi:10.1371/journal.pone.0326534)
Supplement: S1 File — (DOCX) [file pone.0326534.s001.docx]

**Appendix 1. Codes run in TriNetX database**

(Codes were run on December 20^th^, 2023, on the US collaborative Network from TriNetX database, for cohorts building and outcome measurements)

**Establishments of cohorts**

Inclusion criteria:

ICD-10-CM: I50.3 Diastolic (congestive) heart failure

ICD-10-CM: E11 Type 2 diabetes mellitus

ATC: A10BJ Glucagon-like peptide-1 (GLP-1) analogues

ATC: A10BK Sodium-glucose co-transporter 2 (SGLT-2) inhibitors

ICD-10-CM: N18.3 Chronic kidney disease, stage 3 (moderate)

ICD-10-CM: N18.4 Chronic kidney disease, stage 4 (severe)

ICD-10-CM: N18.5 Chronic kidney disease, stage 5

ICD-10-CM: N18.6 End stage renal disease

(N18.3-N18.6 were utilized in subgroup for adjustments of moderate to severe chronic kidney disease)

ICD-10-CM: I25 Chronic ischemic heart disease

(I25 was utilized in subgroup adjustments of chronic ischemic heart disease)

Exclusion criteria and outcome definition:

ICD-10-CM: I50.2 Systolic (congestive) heart failure

ATC: A10BJ Glucagon-like peptide-1 (GLP-1) analogues

ATC: A10BK Sodium-glucose co-transporter 2 (SGLT-2) inhibitors

ICD-10-CM: I21 Acute myocardial infarction

ICD-10-CM: I24 Other acute ischemic heart diseases

ICD-10-CM: I61 Nontraumatic intracerebral hemorrhage

ICD-10-CM: I62 Other and unspecified nontraumatic intracranial hemorrhage

ICD-10-CM: I63 Cerebral infarction

Characteristics and propensity-score matching

ICD-10-CM: I10 Essential (primary) hypertension

ICD-10-CM: I25 Chronic ischemic heart disease

ICD-10-CM: I48 Atrial fibrillation and flutter

ICD-10-CM: I34 Nonrheumatic mitral valve disorders

ICD-10-CM: I35 Nonrheumatic aortic valve disorders

ICD-10-CM: E78 Disorders of lipoprotein metabolism and other lipidemias

ICD-10-CM: I65 Occlusion and stenosis of precerebral arteries, not resulting in cerebral infarction

ICD-10-CM: I67 Other cerebrovascular diseases

ICD-10-CM:I69 Sequelae of cerebrovascular disease

ICD-10-CM: N18.3 Chronic kidney disease, stage 3 (moderate)

ICD-10-CM: N18.4 Chronic kidney disease, stage 4 (severe)

ICD-10-CM: N18.5 Chronic kidney disease, stage 5

ICD-10-CM:N18.6 End stage renal disease

ICD-10-CM: N18.1 Chronic kidney disease, stage 1

ICD-10-CM: N18.2 Chronic kidney disease, stage 2 (mild)

ICD-10-CM: I20 Angina pectoris

(Numeric lab data of systolic blood pressure, diastolic blood pressure, glycated

hemoglobin and body mass index were run with TriNetX curated codes.
