# Supplementary material for: Superior cardiovascular protection with GLP-1 RAs over SGLT2 inhibitors in DM and HFpEF: A propensity score matching study
Source: PLoS One. 2025 Jun 26;20(6):e0326534. doi: 10.1371/journal.pone.0326534 (PMC12200838; doi:10.1371/journal.pone.0326534)
Supplement: Attachment — Submitted filename: PLOS review.docx [file pone.0326534.s003.docx]

This propensity score match study by Li et al. provides valuable insights into the cardiovascular protective effects of GLP-1 receptor agonists compared to SGLT2 inhibitors in patients with HFpEF and diabetes. The use of a large, matched cohort from the TriNetX database adds strength to the findings, and the long-term follow-up offers a comprehensive view of the outcomes. The detailed subgroup analyses, which highlight significant benefits in specific patient populations, further enhance the clinical relevance of the study. The exploration of composite cardiovascular outcomes and specific risks, such as the progression to systolic heart failure and stroke, adds depth to the understanding of these therapies. The slightly diminishing trend over time is also a crucial observation.

However there are certain aspects which further consideration:

**Introduction**

Line 89-98: Overall, the introduction flows well, but it lacks some key details regarding HFpEF and DM, which weakens the rationale for the study. To strengthen the introduction, I recommend including more specific information about the pathophysiology of HFpEF in the context of diabetes and expanding on why a direct comparison of GLP-1 receptor agonists and SGLT2 inhibitors is crucial for this population.

**Discussion**

The discussion is well written; however, the use of headings disrupts the flow and cohesiveness of the text. I recommend removing these headings and rephrasing to enhance continuity between paragraphs, which would create a smoother reading experience.

Line 263-267: *“ This study found that GLP-1 RAs reduced the risk of cardiovascular events, progression to SHF, and stroke compared with SGLT2 inhibitors in patients with HFpEF and DM. The benefits appeared to diminish slightly over the 3-year follow-up. GLP-1 RAs also showed a trend toward reduced MI and mortality, although this was not statistically significant “* should be rephrased to:

Our results demonstrate that GLP-1 receptor agonists provide better cardiovascular protection than SGLT2 inhibitors in patients with HFpEF and DM, significantly reducing the risk of cardiovascular events, progression to SHF, and stroke. While the benefits were strongest in the initial treatment stages, there was a slight reduction in efficacy over the 3-year follow-up. Additionally, GLP-1 RAs showed a trend towards fewer MI’s and lower mortality rates, though these findings were not statistically significant.

Line 270-271: This point has been reiterated in lines 286-288. Since the rationale is already outlined in the introduction (lines 117-121), it is unnecessary to restate it here.

Line 378: “ *However, some limitations of this study warrant further consideration* “.

This starting sentence seems incomplete, it should be rephrased to:

While this propensity score-matched study presents significant findings, it is important to acknowledge some limitations that warrant further consideration.
